# Vitamin D deficiency, depression severity, and suicide risk: evidence from patients with major depressive disorder

**DOI:** 10.55730/1300-0144.6371

**Published:** 2026-07-01

**Authors:** Elif KÜÇÜK, Feride UYSAL, Seda ŞENKARDEŞ KANDEMİR, Özgür ÖZKALAYCI, Canay PAMUKÇU, Nihal TAŞTEKİN, Merih ALTINTAŞ

**Affiliations:** 1 Department of Psychiatry, Faculty of Medicine, Yüksek İhtisas University, Ankara, Turkiye; 2 Department of Psychiatry, Kartal Dr. Lütfi Kırdar City Hospital, University of Health Sciences, İstanbul, Turkiye; 3 Department of Internal Medicine, Bağcılar Training and Research Hospital, İstanbul, Turkiye; 4 Department of Psychiatry, Bağcılar Training and Research Hospital, İstanbul, Turkiye; 5 Department of Psychiatry, Faculty of Medicine, Üsküdar University, İstanbul, Turkiye

**Keywords:** Major depressive disorder, vitamin D, suicidal ideation, depression severity

## Abstract

**Background/aim:**

Major depressive disorder (MDD) is a common psychiatric condition characterized by depressive symptoms and suicide-related clinical features. Vitamin D status has been investigated as a potential clinical correlate of depression and suicidal ideation, but the direction and specificity of these associations remain uncertain. This cross-sectional study aimed to examine the associations among serum 25-hydroxyvitamin D [25(OH)D] levels, depressive symptom severity, suicidal ideation, and suicide-related cognitive–affective dimensions in adults diagnosed with MDD.

**Materials and methods:**

This cross-sectional study included 97 adults aged 18–65 years who were diagnosed with MDD according to DSM-5 criteria. Depressive symptom severity and suicide-related outcomes were assessed using the Hamilton Depression Rating Scale (HDRS), Suicidal Ideation Scale (SIS), and Suicide Probability Scale (SPS). Vitamin D status was classified as deficient (<20 ng/mL), insufficient (20–29 ng/mL), or sufficient (≥30 ng/mL). Associations were examined using multivariable generalized linear models adjusted for available sociodemographic and clinical covariates.

**Results:**

Vitamin D deficiency was observed in 61.9% of participants and the mean serum 25(OH)D level was 18.4 ± 11.9 ng/mL. Lower serum 25(OH)D levels were significantly associated with higher HDRS, SIS, and SPS total/subscale scores. In adjusted models, higher serum 25(OH)D levels remained inversely associated with HDRS scores, SIS scores, and total SPS scores. Parental status was also associated with lower SIS scores, while a history of suicide attempts was associated with higher SPS–Suicidal Ideation scores.

**Conclusion:**

Lower serum 25(OH)D levels were associated with greater depressive symptom severity and higher suicide-related scale scores in adults with MDD, particularly across cognitive–affective domains relevant to suicide probability. However, given the study’s cross-sectional single-center design, absence of a healthy control group, modest sample size, and adjustment limited to available covariates, these findings should be interpreted as associations rather than evidence of causality, directionality, or established biomarker utility.

## 1. Introduction

Major depressive disorder (MDD) is a prevalent psychiatric condition characterized by depressed mood, anhedonia, feelings of worthlessness, and suicidal ideation [1]. Recent global estimates indicate that approximately 332 million individuals are affected by depression worldwide, corresponding to nearly 4% of the total population and 5.7% of adults, with higher prevalence among women than men [2]. MDD substantially impairs social and occupational functioning, reduces quality of life, complicates the management of chronic medical conditions, and contributes to suicide-related mortality and socioeconomic burden [3–5]. In this context, early identification of clinical and biological correlates associated with depressive severity and suicide-related features remains an important research priority [6,7].

The pathophysiology of depression is multifactorial and involves interactions among genetic, psychological, environmental, immune, and biological processes. Increasing evidence suggests that immune dysregulation, particularly alterations in proinflammatory cytokine activity, may be involved in depression and suicide-related outcomes [8–10]. Consistent with this framework, Serafini et al. reported that neuroinflammatory processes may be associated with suicide risk, with alterations in inflammatory markers observed in peripheral blood, cerebrospinal fluid, and the central nervous system among individuals at risk for suicide [11]. Postmortem findings have also shown increased vitamin D receptor gene expression in brain tissues of individuals who died by suicide compared to controls, suggesting a possible relationship between vitamin D-related pathways and suicide-related biology [12].

Vitamin D has been increasingly examined as a potential biological correlate of depression because of its role in neurological and immunological regulation. Vitamin D insufficiency may be associated with depressive symptoms through pathways involving cholinergic, dopaminergic, and noradrenergic neurotransmission [13]. As a neuroactive steroid, vitamin D is involved in brain development, neurotransmission, neuroprotection, and immune response modulation [14]. Vitamin D receptors and 1α-hydroxylase enzymes are expressed in brain regions implicated in mood regulation, including the prefrontal cortex, hippocampus, amygdala, and substantia nigra [15,16].

Several epidemiological studies have reported inverse associations between serum 25-hydroxyvitamin D [25(OH)D] levels and the prevalence or severity of depressive symptoms [17,18]. Some randomized controlled trials and metaanalyses have suggested that vitamin D supplementation may improve depressive symptoms; however, findings remain inconsistent and may vary according to baseline vitamin D status, dose, duration of supplementation, and sample characteristics [19,20]. In a large cohort study, Kim et al. reported an association between vitamin D deficiency and increased suicidal ideation [21]. A recent systematic review further suggested that vitamin D deficiency may be related to inflammatory regulation and serotonin synthesis, both of which have been implicated in depression and suicide-related outcomes [22].

Despite growing interest in vitamin D status in relation to depression and suicide-related outcomes, important gaps remain. Previous studies have mainly focused on overall depressive symptom severity or suicidal ideation as a single outcome, whereas specific cognitive–affective dimensions of suicide probability, including hopelessness, negative self-evaluation, suicidal ideation, and hostility, have been less extensively examined. Moreover, combined evaluations of serum 25(OH)D levels, depressive severity, suicide-related scale scores, and psychosocial factors within a clinically diagnosed MDD sample remain limited. Accordingly, the present study aimed to examine the associations among serum 25(OH)D levels, depressive symptom severity, suicidal ideation, and suicide-related cognitive–affective dimensions in adults with MDD, interpreting the findings within an associative rather than causal framework.

## 2. Materials and methods

### 2.1. Study design and participants

The study was conducted between December 2022 and December 2024 at Kartal Dr. Lütfi Kırdar City Hospital (İstanbul, Türkiye) and included 97 adults aged 18–65 years who were diagnosed with MDD according to the criteria of the Diagnostic and Statistical Manual of Mental Disorders, Fifth Edition (DSM-5). Participants were consecutively recruited from eligible patients presenting to the psychiatry outpatient clinic. Patients who did not meet the inclusion criteria, met any exclusion criterion, declined participation, or had incomplete essential clinical or laboratory data were not included. The final analytical sample consisted of 97 participants with complete serum 25(OH)D measurements and psychiatric scale assessments.

The diagnosis of MDD was established by an experienced psychiatrist through a face-to-face DSM-5-based clinical interview. A standardized structured diagnostic interview, such as the Structured Clinical Interview for DSM-5 or Mini-International Neuropsychiatric Interview, was not administered. The diagnostic evaluation included current depressive symptoms, psychiatric and treatment history, and possible comorbid psychiatric conditions. Bipolar disorder, psychotic disorders, substance use disorder, and other psychiatric disorders that could confound the clinical evaluation were assessed through clinical interviews and reviews of available clinical records when necessary. Interrater reliability analysis was not performed because diagnoses were established by the evaluating psychiatrist in routine clinical practice, and cases with diagnostic uncertainty were excluded.

Serum 25(OH)D measurement and psychiatric scale assessments were performed within the same clinical evaluation period. Blood sampling and psychometric assessments were completed during the same outpatient assessment process to ensure temporal consistency between biochemical and clinical data.

The inclusion criteria were age between 18 and 65 years, a DSM-5 diagnosis of MDD, and provision of written informed consent. The exclusion criteria were psychotic depression, bipolar disorder, schizophrenia, substance use disorder, other psychiatric disorders, pregnancy or lactation, use of vitamin D or other immunomodulatory agents within the preceding 3 months, and chronic systemic diseases, including autoimmune or endocrine disorders.

All participants were informed about the study and written informed consent was obtained before clinical assessment. The study was approved by the Ethics Committee of Kartal Dr. Lütfi Kırdar City Hospital (approval number: 2022/514/237/4; date: November 14, 2022) and was conducted in accordance with the Declaration of Helsinki. A priori power analysis based on a multivariable regression framework with five predictors indicated that at least 92 participants were required, assuming an α error of 0.05, power of 0.80, and medium effect size (f^2^ = 0.15). A medium effect size (f^2^ = 0.15) was selected a priori based on conventional Cohen benchmarks given the limited availability of directly comparable studies and the heterogeneity of previously reported effect estimates in research on vitamin D status, depressive symptoms, and suicide-related outcomes.

### 2.2. Sociodemographic and clinical data form

Sociodemographic and clinical characteristics were recorded using a study-specific data form completed for each participant. The form included age, sex, educational attainment, marital status, economic status, parental status, psychiatric treatment history, history of suicide attempts, family history of suicide attempts, and history of psychosocial crises. When necessary, supplementary interviews with participants’ relatives were conducted to clarify missing or ambiguous sociodemographic information.

Although psychiatric treatment history and key sociodemographic and clinical variables were recorded, data on body mass index (BMI), smoking, alcohol consumption, physical activity, sunlight exposure, seasonal variation, detailed dietary intake, sleep disorders, antidepressant treatment characteristics, illness duration, and MDD episode characteristics were not systematically available in the original dataset and therefore could not be incorporated into the clinical characterization or adjusted analyses.

### 2.3. 25-Hydroxyvitamin D3 levels

Peripheral venous blood samples were collected from all participants in the morning between 08:30 and 10:00 after 6–10 h of fasting. Samples were obtained into plain tubes, allowed to clot at room temperature, and centrifuged at 4000 rpm for 10 min. Serum was separated and stored at −80 °C until analysis. Serum 25(OH)D levels were measured using liquid chromatography–tandem mass spectrometry (LC-MS/MS) on a Waters Quattro Premier XE system (Waters Corp., Milford, MA, USA). All measurements were performed in the same clinical biochemistry laboratory using the same analytical platform. Calibration and quality-control procedures followed the manufacturer’s instructions and routine laboratory protocols, and intraassay and interassay coefficients of variation were within the laboratory’s acceptable analytical performance limits. Blood samples were collected between December 2022 and December 2024 without seasonal stratification; therefore, seasonality was not included as an adjustment variable. Serum 25(OH)D levels were classified as deficient (<20 ng/mL), insufficient (20–29 ng/mL), or sufficient (≥30 ng/mL) [23].

### 2.4. Hamilton Depression Rating Scale

The Hamilton Depression Rating Scale (HDRS), developed by Hamilton in 1960, is widely used to assess depressive symptom severity [24]. The 17-item version evaluates symptoms such as depressed mood, guilt, suicidal ideation, insomnia, anxiety, somatic symptoms, and insight. Items are scored on scales of 0–2 or 0–4, yielding a total score of 0–53, with higher scores indicating greater depressive severity. Total scores of 0–7 indicate no depression, 8–15 mild depression, 16–28 moderate depression, and ≥29 severe depression. The Turkish version has demonstrated strong validity and reliability, with interrater consistency values ranging from 0.87 to 0.98 [25].

### 2.5. Suicidal Ideation Scale

The Suicidal Ideation Scale (SIS) is a 17-item self-report instrument developed to assess the presence and severity of suicidal ideation and related behaviors [26]. It evaluates suicidal ideation, planning, previous attempts, and related clinical indicators. Scores are interpreted together with clinical information, with higher scores indicating greater severity of suicidal ideation. The Turkish version has demonstrated strong validity and reliability, with high internal consistency (Cronbach alpha = 0.91) [27].

### 2.6. Suicide Probability Scale

The Suicide Probability Scale (SPS), developed by Cull and Gill, is a 36-item, four-point Likert-type self-report instrument designed to assess suicide risk in adolescents and adults [28]. It includes four subscales: hopelessness, suicidal ideation, negative self-evaluation, and hostility. Total scores range from 36 to 144, with higher scores indicating greater suicide probability. The Turkish adaptation and validity–reliability study were conducted by Atlı et al. [29].

### 2.7. Statistical analysis

Continuous variables are presented as mean ± standard deviation or median (interquartile range), depending on distributional characteristics, and categorical variables as number (percentage). Associations were assessed using Pearson r correlations for normally distributed or appropriately transformed variables and the Kendall tau for nonnormally distributed or ordinal variables. Bayesian Kendall tau analyses were performed as complementary analyses to quantify the strength of evidence for or against associations using Bayes factors (BF_10_), with default stretched beta prior width of 1 in the open-source JAMOVI jsq module.

Candidate predictors identified in preliminary analyses and clinically relevant variables were entered into generalized linear models. Separate models were fitted for HDRS total score, SIS score, SPS subscale scores, and SPS total score. In each model, the psychiatric scale score was specified as the dependent variable and serum 25(OH)D level was entered as the main continuous independent variable per 1 ng/mL increase. Sociodemographic and clinical covariates were included as appropriately coded continuous or binary/dummy variables. Overdispersion was assessed using Pearson chi-square/degrees of freedom and deviance/degrees of freedom statistics. Because overdispersion was present, overdispersed Poisson models were used to obtain robust standard errors and confidence intervals (CIs). Model diagnostics included assessment of residuals, influential observations, and multicollinearity using variance inflation factors.

BF_10_ values of 1–3, 3–10, 10–30, 30–100, and >100 were interpreted as anecdotal, moderate, strong, very strong, and extreme evidence of an association, respectively. Values below 1 were interpreted as evidence favoring the absence of an association. Holm correction was applied where appropriate to account for multiple comparisons, and corrected p values are reported. No missing data were present in the final analytical dataset; therefore, no imputation was performed. Analyses were conducted using open-source JAMOVI version 2.3.18 with the jsq and GAMLj modules.

## 3. Results

The study comprised 97 participants, including 70 women (72.2%) and 27 men (27.8%). The mean age was 33.6 ± 10.2 years and the mean serum 25(OH)D concentration was 18.4 ± 11.9 ng/mL. According to the prespecified cut-off values, 61.9% (n = 60) of participants had vitamin D deficiency, 25.8% (n = 25) had insufficiency, and 12.4% (n = 12) had sufficiency. Overall, 55.7% (n = 54) were married, 54.6% (n = 53) had children, 11.3% (n = 11) reported a history of suicide attempts, 13.4% (n = 13) reported a family history of suicide attempts, and 55.7% (n = 54) reported at least one lifetime psychosocial crisis (Table 1).

Serum 25(OH)D levels were significantly and inversely correlated with HDRS, SIS, and SPS total/subscale scores (Table 2). The strongest association was observed for total SPS scores, indicating that lower vitamin D levels were associated with higher depressive symptom severity and higher suicide-related scale scores.

Exploratory associations between psychiatric scale scores and sociodemographic or clinical variables are presented in Table 3. HDRS total scores were not significantly associated with the evaluated sociodemographic variables. Marital status and parental status were associated with lower SIS scores. No meaningful exploratory associations were observed between sex, educational level, psychiatric treatment history, history of suicide attempts, or family history of suicide attempts and HDRS, SIS, or SPS scores. Weak associations between psychosocial crisis history and HDRS or SPS subscales were not supported by strong Bayes factors.

**Figure 1:**
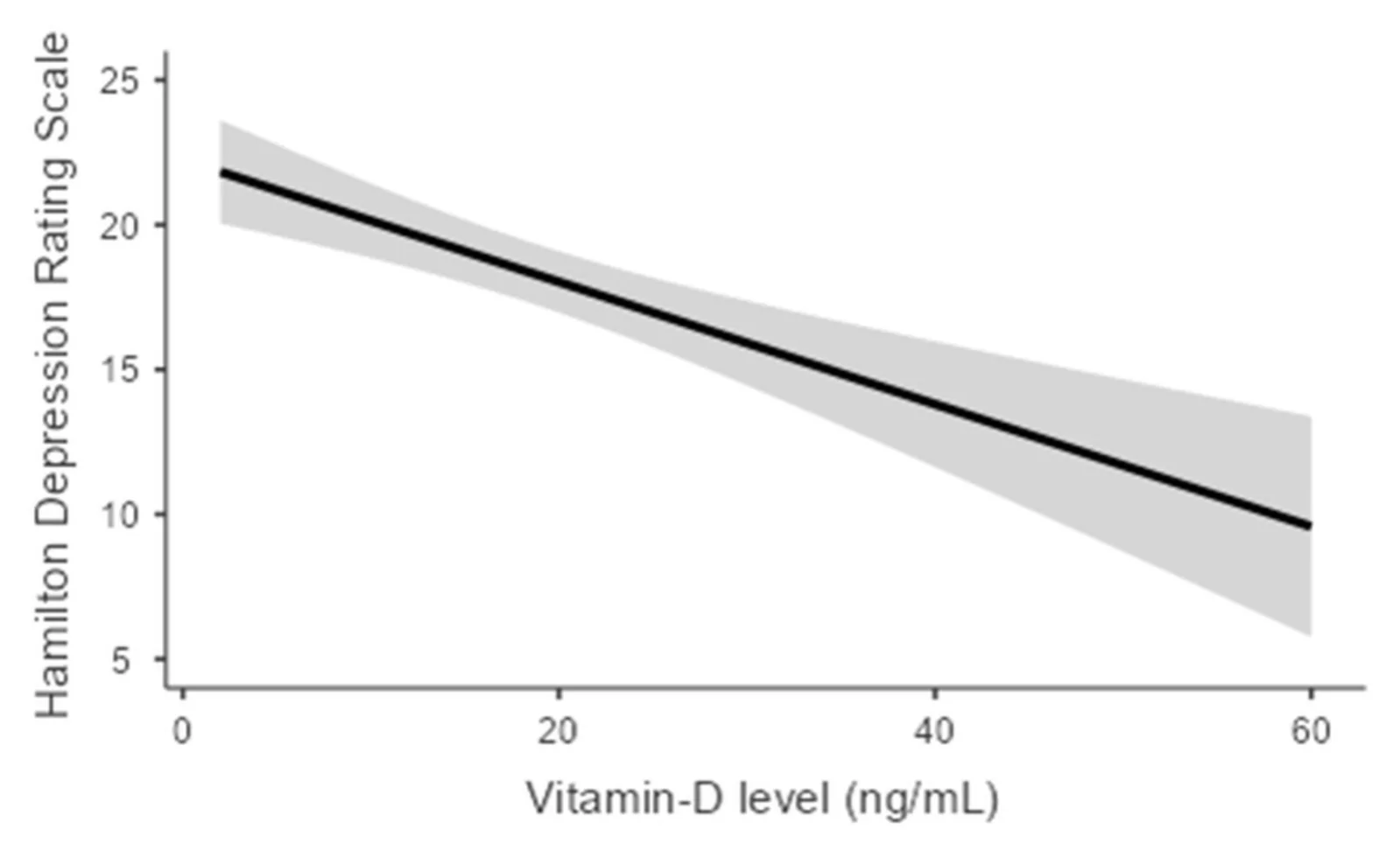
Model-based association between serum 25(OH)D level and HDRS score.

**Figure 2:**
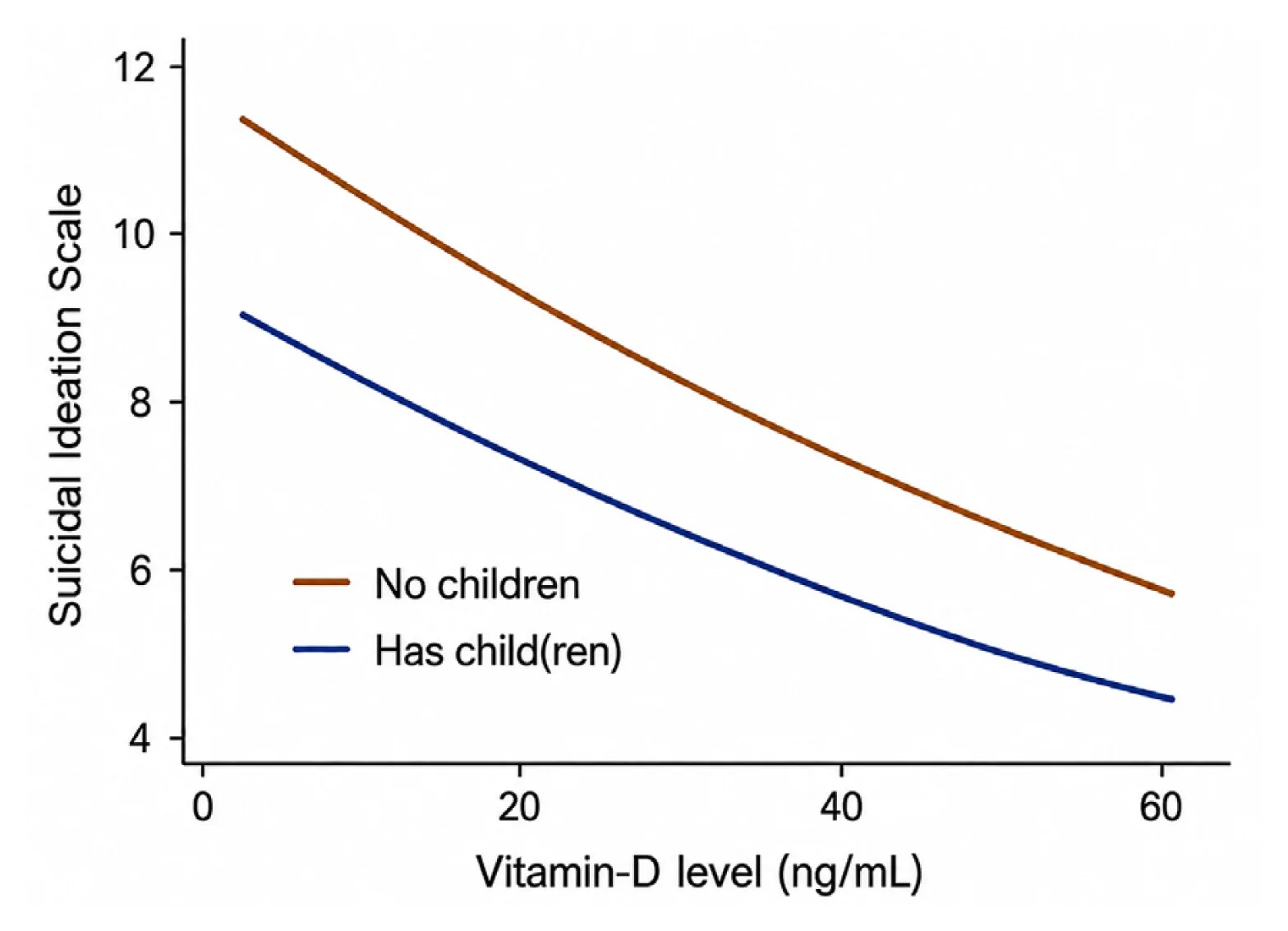
Model-based association between serum 25(OH)D level and SIS score.

In multivariable generalized linear models adjusted for available sociodemographic and clinical covariates, serum 25(OH)D levels remained inversely associated with depressive symptom severity and suicide-related scale scores. Model-based fitted associations for the HDRS and SIS are shown in Figures 1 and 2, respectively. Each 1 ng/mL increase in serum 25(OH)D was associated with lower expected HDRS scores (exp[β] = 0.987; 95% CI: 0.982–0.992; model R^2^ = 0.219) and lower expected SIS scores (exp[β] = 0.989; 95% CI: 0.981–0.996). Not having children was associated with higher expected SIS scores (exp[β] = 1.26; 95% CI: 1.07–1.48; Holm-adjusted p = 0.006; model R^2^ = 0.145).

**Figure 3:**
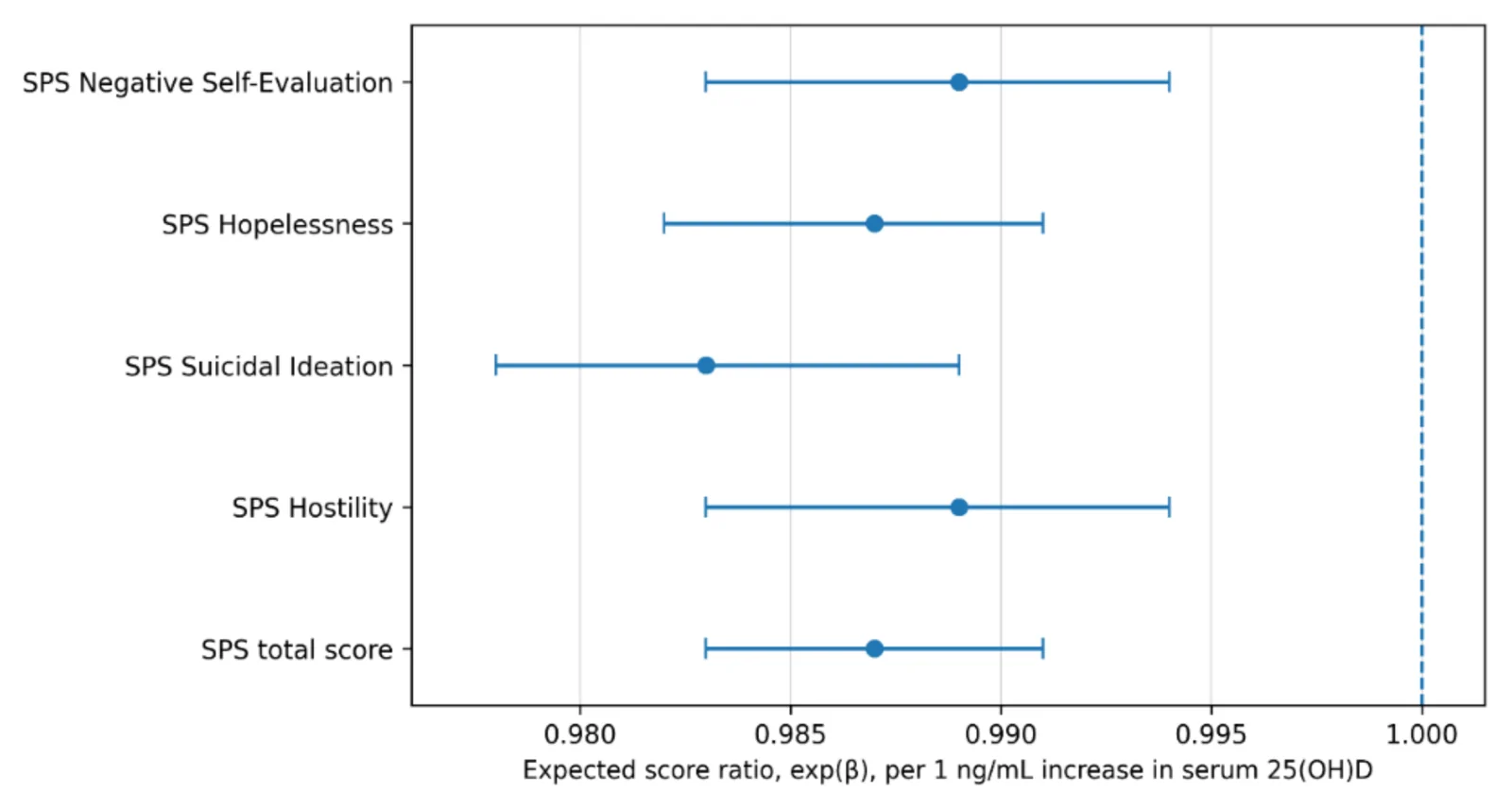
Generalized linear model results for SPS outcomes.

In separate generalized linear models for SPS outcomes, each 1 ng/mL increase in serum 25(OH)D was associated with lower expected scores for negative self-evaluation (exp[β] = 0.989; 95% CI: 0.983–0.994), hopelessness (exp[β] = 0.987; 95% CI: 0.982–0.991), suicidal ideation (exp[β] = 0.983; 95% CI: 0.978–0.989), hostility (exp[β] = 0.989; 95% CI: 0.983–0.994), and total SPS score (exp[β] = 0.987; 95% CI: 0.983–0.991) (Figure 3). In the SPS–Suicidal Ideation model, absence of suicide attempt history was associated with lower expected scores (exp[β] = 0.829; 95% CI: 0.694–0.998; Holm-adjusted p = 0.043). The association between parental status and SPS–Hostility did not remain statistically significant after Holm correction (Holm-adjusted p = 0.061) and was interpreted cautiously.

## 4. Discussion

The present study showed inverse associations between serum 25(OH)D levels and depressive symptom severity, suicidal ideation, and suicide-related cognitive–affective dimensions in adults with MDD. Lower serum 25(OH)D levels were associated with higher HDRS, SIS, and total/subscale SPS scores, particularly in the domains of negative self-evaluation, hopelessness, suicidal ideation, and hostility. In addition, a history of suicide attempts was associated with higher SPS–Suicidal Ideation scores, whereas marital and parental status were associated with lower suicide-related scores. Overall, these findings suggest that vitamin D status may be relevant to suicide-related clinical features in MDD but should be interpreted within a broader psychosocial and clinical framework.

The observed inverse associations are broadly consistent with previous observational studies reporting lower serum 25(OH)D levels in relation to greater depressive symptom burden across different populations [30–32]. The present study extends this literature by examining not only overall depressive severity but also specific suicide-related cognitive and affective dimensions. The association of lower 25(OH)D levels with negative self-evaluation, hopelessness, suicidal ideation, and hostility is also consistent with prior evidence linking vitamin D status to hostility and psychosocial stress [33]. Nevertheless, the specificity and direction of these associations remain uncertain because of the study’s cross-sectional design.

Evidence from preventive, interventional, and genetically informed studies remains more limited and inconsistent. Although metaanalytic data suggest that vitamin D supplementation may be associated with improvement in depressive symptoms in selected populations, the magnitude of benefit appears to vary according to baseline 25(OH)D status, dose, duration of supplementation, and sample characteristics [34]. In contrast, a two-sample Mendelian randomization study found no evidence for a causal relationship between 25(OH)D levels and MDD or a broader internalizing psychopathology dimension [35]. Similarly, a 2024 Mendelian randomization study using UK Biobank data reported no overall linear association between genetically predicted 25(OH)D levels and probable lifetime major depression, although nonlinear analyses suggested a possible association confined to individuals in the lowest 25(OH)D quintile [36]. Therefore, the present findings should be regarded as observational associations rather than evidence that vitamin D supplementation would reduce depressive severity or suicide-related symptoms in patients with MDD.

With regard to suicide-related outcomes, our findings are in line with clinical studies reporting lower 25(OH)D levels among individuals with suicide attempts or self-harm, as well as with recent metaanalytic evidence linking vitamin D deficiency to suicidal ideation, suicide attempts, and related behaviors [37–41]. However, the evidence remains heterogeneous. For example, a cohort study in Chinese adolescents reported a nonlinear association between 25(OH)D levels and suicidal ideation in female participants, whereas Mendelian randomization analyses did not support a causal relationship, suggesting that confounding or reverse causality may partly explain the observed association [40]. Thus, the present results should be interpreted as clinical correlations rather than evidence of causality or diagnostic specificity.

The high prevalence of vitamin D deficiency in the present sample may have contributed to the observed associations, consistent with the view that vitamin D-related clinical associations may be more apparent among individuals with marked deficiency. In line with previous evidence linking lower 25(OH)D levels to depressive symptoms, hopelessness, and psychosocial stress, higher vitamin D levels in our sample were associated with lower SIS and SPS scores, particularly across the negative self-evaluation, hopelessness, and suicidal ideation subscales [33,42]. The association between previous suicide attempts and higher SPS–Suicidal Ideation scores is consistent with evidence identifying prior suicidal behavior as a major clinical indicator of subsequent suicide-related outcomes [43,44]. In parallel, the associations of marital and parental status with lower suicide-related scores are consistent with population-based findings emphasizing the protective relevance of family bonds and social connectedness [45,46]. These findings support a multidimensional interpretation in which vitamin D status is considered not as an isolated biological determinant but rather as a potentially relevant clinical correlate alongside psychosocial resources and established suicide-related risk factors.

The nonsignificant associations observed for several sociodemographic and clinical variables also require cautious interpretation. In this sample, sex, educational level, psychiatric treatment history, and family history of suicide attempts were not significantly associated with depressive severity or suicide-related scale scores. These findings may reflect limited statistical power, the predominance of female participants, and the relative clinical homogeneity of the sample rather than the absence of clinically meaningful relationships. Previous evidence suggests that the influence of demographic and clinical factors on suicide-related outcomes may vary according to sample composition, illness severity, sociocultural context, and the specific construct assessed [44,46]. Similarly, although history of suicide attempts was not consistently associated with all suicide-related outcomes in bivariate analyses, it remained associated with higher SPS–Suicidal Ideation scores in subscale-specific models, supporting its clinical relevance as a risk indicator.

Several limitations of this study should be acknowledged. First, the cross-sectional design precludes causal inference and does not exclude reverse causality; depressive or suicide-related symptoms may reduce sunlight exposure, physical activity, nutritional quality, and self-care, thereby contributing to lower vitamin D levels. Second, the absence of a healthy control group limits conclusions regarding the specificity of the findings to MDD and suicide-related outcomes, particularly given the high background prevalence of vitamin D deficiency in Türkiye. A nationwide metaanalysis reported vitamin D deficiency rates of approximately 63% in the general population and 63.5% among adults [47]. Third, several important determinants of vitamin D status, including BMI, smoking, alcohol consumption, physical activity, sunlight exposure, seasonality, detailed dietary intake, sleep disorders, antidepressant treatment characteristics, illness duration, and MDD episode features, were not systematically available. Therefore, the regression findings should be interpreted as associations adjusted for available covariates rather than as evidence of independence from all relevant confounders. Finally, the single-center design, modest sample size, predominance of female participants, and geographically restricted recruitment may limit the generalizability. Although the sample size met the a priori requirement for the primary regression framework, the use of multiple models, several outcomes, and SPS subdomains may increase the risk of model instability and reduce the precision of some estimates. Future controlled, multicenter, and longitudinal studies with more comprehensive assessment of lifestyle, seasonal, clinical, and treatment-related variables are needed.

In conclusion, lower serum 25(OH)D levels were associated with greater depressive symptom severity and higher suicide-related scale scores in adults with MDD, particularly across the cognitive–affective domains of suicidal ideation, hopelessness, and negative self-evaluation. The associations of marital and parental status with lower suicide-related scores further underscore the importance of incorporating psychosocial factors into clinical assessment. Nevertheless, given the cross-sectional and single-center design, absence of a healthy control group, modest sample size, limited number of participants with a history of suicide attempts, and adjustment limited to available covariates, these findings should be interpreted as associations within a clinical MDD sample rather than as evidence of causality, disease specificity, or established biomarker utility. Further controlled and longitudinal studies are required to clarify the direction, specificity, and clinical relevance of these associations.

## Figures and Tables

**Table 1:** Sociodemographic and clinical characteristics of the participants.

		n (%)
**Sex**	Female	70 (72.2 %)
	Male	27 (27.8 %)
**Marital status**	Married	54 (55.7 %)
	Single	43 (44.3 %)
**Having a child**	Yes	53 (54.6 %)
	No	44 (45.4 %)
**Psychiatric treatment history**	Yes	38 (39.2 %)
	No	59 (60.8 %)
**History of suicide attempts**	Yes	11 (11.3 %)
	No	86 (88.7 %)
**Family history of suicide attempts**	Yes	13 (13.4 %)
	No	84 (86.6 %)
**Psychosocial crisis**	Yes	54 (55.7 %)
	No	43 (44.3 %)

**Table 2:** Correlations between serum vitamin D levels and psychiatric scales.

	Vitamin D levels^P^		Vitamin D status^K^	
	r	p	r	p
**HDRS total**	−0.453^***^	<0.001	−0.376^***^	<0.001
**Suicidal Ideation Scale**	−0.285^**^	0.007	−0.199^*^	0.017
**SPS-negative self-evaluation**	−0.393^***^	<0.001	−0.357^***^	<0.001
**SPS-SI TOTAL**	−0.529^***^	<0.001	−0.469^***^	<0.001
**SPS-hopelessness Total**	−0.499^***^	<0.001	−0.459^***^	<0.001
**SPS-hostility Total**	−0.369^***^	<0.001	−0.369^***^	<0.001
**SPS -total**	−0.537^***^	<0.001	−0.458^***^	<0.001

HDRS: Hamilton Depression Rating Scale; SIS: Suicidal Ideation Scale; SPS: Suicide Probability Scale; NSE: negative self-evaluation; SI: suicidal ideation. Associations are presented as Pearson r and Kendall tau coefficients. Nonnormally distributed continuous variables were transformed prior to Pearson r analysis. Vitamin D status was analyzed as an ordinal variable based on categories of deficiency, insufficiency, and sufficiency.

**Table 3:** Exploratory associations of sociodemographic and clinical variables with psychiatric scale scores.

	HDRS		SIS		SPS- NSE		SPS-SI		SPS-hopelessness		SPS-hostility		SPS total		Vitamin D level	
	r	BF_10_	r	BF_10_	r	BF_10_	r	BF_10_	r	BF_10_	r	BF_10_	r	BF_10_	r	BF_10_
**Sex**	0.091	0.314	0.015	0.136	0.143	0.161	0.042	0.160	0.055	0.182	0.040	0.156	0.052	0.175	0.013	0.140
**Marital status**	−0.038	0.153	0.200	8.384	0.086	0.286	0.030	0.145	−0.056	0.183	0.114	0.515	0.030	0.146	0.075	0.237
**Having a child**	0.013	0.135	−0.232	35.932	0.095	0.340	0.074	0.234	0.034	0.150	0.158	1.778	0.094	0.331	0.041	0.161
**Education**	0.067	0.211	0.081	0.263	0.122	0.619	−0.074	0.232	0.033	0.148	−0.041	0.157	−0.013	0.135	0.084	0.271
**Psychiatric treatment history**	0.085	0.282	−0.025	0.142	0.061	0.196	0.006	0.133	−0.100	0.375	−0.050	0.172	−0.036	0.151	0.025	0.146
**History of suicide attempts**	0.032	0.147	0.123	0.647	0.117	0.551	0.151	1.413	0.021	0.139	0.107	0.436	0.110	0.471	0.043	0.164
**Family history of suicide attempts**	0.084	0.276	0.027	0.143	0.016	0.136	0.056	0.184	0.042	0.159	0.150	1.361	0.067	0.211	−0.080	0.253
**Psychosocial crisis**	0.132	0.811	−0.018	0.137	0.111	0.481	0.052	0.176	0.125	0.668	0.124	0.651	0.123	0.637	0.013	0.140

HDRS: Hamilton Depression Rating Scale; SIS: Suicidal Ideation Scale; SPS: Suicide Probability Scale; NSE: negative self-evaluation; SI: suicidal ideation. Exploratory associations are presented as Kendall tau coefficients with Bayes factors (BF_10_). Analyses involving nominal or dichotomous variables should not be interpreted as conventional correlations between two continuous variables. Bayesian analyses were used only as complementary evidence for association testing and were not derived from generalized linear models.

## Data Availability

The data that support the findings of this study are available on request from the corresponding author. The data are not publicly available due to privacy or ethical restrictions.
